# A Nomogram for the Prediction of Progression and Overall Survival in Childhood Acute Lymphoblastic Leukemia

**DOI:** 10.3389/fonc.2020.01550

**Published:** 2020-08-25

**Authors:** Dan Zhang, Yu Cheng, Jia Fan, Juan Yao, Zijun Zhao, Yao Jiang, Yiqin Li, Zhihua Zuo, Yan Tang, Yongcan Guo

**Affiliations:** ^1^Clinical Laboratory of Traditional Chinese Medicine Hospital Affiliated to Southwest Medical University, Luzhou, China; ^2^Sichuan Luzhou Traditional Chinese Medicine Hospital, Luzhou, China; ^3^Southwest Medical University, Luzhou, China; ^4^Department of Clinical Laboratory Medicine, Jinniu Maternity and Child Health Hospital of Chengdu, Chengdu, China

**Keywords:** acute lymphoblastic leukemia, prognostic factor, nomogram, overall survival, prognosis

## Abstract

**Background:** Advances in treatment and supportive care have significantly improved the overall survival (OS) of pediatric patients with acute lymphoblastic leukemia (ALL). However, there is a large number of these patients who continue to relapse after receiving standard treatment. Accurate identification of patients at high risk of relapse and targeted therapy may significantly improve their prognosis. Therefore, the aim of this study was to identify significant prognostic factors for pediatric ALL and establish a novel nomogram for the prediction of survival.

**Methods:** The ALL clinical data of Phases I and II of the Therapeutic Applicable Research to Generate Effective Treatments (TARGET) project were merged and randomly divided into training and validation groups. The LASSO regression model was used to select the specific factors related to the OS of the training group and generate prognostic nomograms according to the selected characteristics. The predictive accuracy of the nomogram for OS was verified using the concordance index of the training and validation groups, the area under the receiver operating characteristic curve for prognostic diagnosis, and the calibration curve.

**Results:** A total of 1,000 children with ALL were included in the TARGET project. Of those, 489 patients had complete follow-up data for further analysis. The data were randomly divided into the training group (*n* = 345) and the validation group (*n* = 144). Seven clinical characteristics, namely age at diagnosis, peripheral white blood cells, bone marrow and CNS site of relapse, ETV6/RUNX1 fusion, TCF3/PBX1, and BCR/ABL1 status, were selected to construct the nomogram. The concordance indices of the training and validation groups were 0.809 (95% confidence interval: 0.766–0.852) and 0.826 (95% confidence interval: 0.767–0.885), respectively. The areas under the receiver operating characteristic curve of the 3-year, 5-year, and 10-year OS in the training group were 0.804, 0.848, and 0.885, respectively, while that of the validation group were 0.777, 0.825, and 0.863, respectively. Moreover, the calibration curves demonstrated a favorable consistency between the predicted and actual survival probabilities.

**Conclusions:** Independent predictors of OS in children with ALL included age at diagnosis, white blood cells, bone marrow site of relapse, CNS site of relapse, ETV6/RUNX1 fusion, TCF3/PBX1, and BCR/ABL1 status. The nomograms developed using these high-risk factors can more simply, accurately, and quantitatively predict the survival of children, and improve treatment and prognosis.

## Introduction

Acute lymphoblastic leukemia (ALL) is the most common malignancy in children. It mainly originates from B-lineage or T-lineage lymphoid progenitor cells. Leukemic cells proliferate abnormally and aggregate in the bone marrow. Consequently, they inhibit normal hematopoiesis, leading to anemia, thrombocytopenia, and neutropenia. In addition, the cells can invade extramedullary tissues, such as the meninges, gonads, thymus, liver, spleen, or lymph nodes, and bone tissue, causing the corresponding lesions. Unlike adult ALL, childhood ALL usually refers to precursor leukemia, which is not unique in terms of immunophenotype; however, it exhibits unique cytogenetic characteristics, as well as unique treatment options and prognosis (1). In recent years, the efficacy of treatment against ALL has significantly improved, and the 5-year survival rate can reach >80% (2, 3). However, there is a large number of children with ALL who continue to relapse after receiving standard treatment and are associated with a low survival rate after relapse (3). ALL consists of multiple entities with different genetic variations, clinical features, and therapeutic responses; it is treated with the same regimen, and patient survival is heterogeneous. Therefore, identifying patients at high risk with a uniform prognosis is critical for effective clinical practice, decision making, and clinical trials.

In the 1950s, Farber and Diamond firstly demonstrated that chemotherapy could induce remission of cancer in humans. The treatment of childhood ALL provides such an example. Once the treatment was determined, the age at diagnosis and white blood cells (WBC) were used as significant prognostic factors to predict efficacy and prognosis, and improve the treatment effect on children with ALL. Over the past decade, studies using gene expression, changes in DNA copy number, and microarray analysis through next-generation sequencing have provided essential insights into the pathogenesis and clinical behavior of ALL (4–6). For example, the detection of the leukemia-associated phenotype by polymerase chain reaction amplification or flow cytometry to determine minimal residual disease may also be the strongest predictor of disease-free survival and overall survival (OS) (7–10). However, owing to the lack of multicenter, large-sample clinical studies, many of the reported prognostic factors for early treatment response have not been compared; thus, it is challenging to translate these data into robust results (11). Therefore, there is an urgent need to comprehensively analyze prognostic factors and develop a model for the accurate prediction of the survival rate of a single child with ALL. This approach will guide the personalized treatment of children with ALL based on different risk factors.

In recent years, numerous studies investigated the prognostic risk factors of childhood ALL. Those research played a vitally important role in reassessing the prognostic risk of children, determining the appropriate treatment intensity, and improving the treatment effect and prognosis (12). However, there is no uniform standard for the assessment of ALL risk. Most previous guidelines were based on the age at diagnosis, peripheral WBC counts, extramedullary leukemia status, tumor cytogenetic characteristics, and treatment response (13, 14). These prognostic risk factors had no commonality in clinical use due to regional and ethnic differences; thus, it was difficult to draw convincing conclusions.

Nomograms are widely used in the field of oncology as prognostic tools for predicting the likelihood of disease. Their easy-to-understand numbers integrate related variables into complex mathematical models. Nomograms can improve the accuracy of discriminant predictions and have been successfully used to quantify the risk of various malignancies (15). However, a nomogram for children with ALL has not been developed thus far. This study used Therapeutic Applicable Research to Generate Effective Treatments (TARGET) data of the National Cancer Center (https://ocg.cancer.gov/programs/target/data-matrix) to develop a nomogram for estimating the individualized prognosis of childhood ALL with 3-, 5-, and 10-year OS, which is the fundamental clinical value for guiding individualized treatment of cancer.

## Materials and Methods

### Data Source and Study Design

We downloaded cohort and analysis data of children with ALL obtained at two different significant stages (Phases I and II) from the TARGET project database (https://ocg.cancer.gov/). The inclusion criterion was the diagnosis of pediatric ALL from 2005 to 2016. The exclusion criteria were as follows: unknown age at diagnosis, uncertain gender, uncertain race, unknown WBC at diagnosis, unknown central nervous system (CNS) status at diagnosis, unknown bone marrow site of relapse, unknown CNS site of relapse, unknown testes site of relapse, unknown ETV6/RUNX1 fusion status, unknown TRISOMIES status, unknown MLL status, unknown TCF3/PBX1 status, and unknown BCR/ABL1 status. Furthermore, we merged the data of all patients in Phases I and II and randomly divided the data into training and validation groups using the R software.

### Cut-Point Optimization of Characteristics

In terms of clinical characteristics, age and peripheral WBC are continuous variables, and the correlation with OS outcomes is not linear. Determining the optimal cut-point is challenging. For the accurate prediction of the prognosis of pediatric ALL in the subsequent analysis, we used the X-tile software (version 3.6.1; Yale University, New Haven, CT, USA). Through this approach, we selected the optimal cutoff value for the two aforementioned continuous variables (16).

### LASSO Regression Analysis

Cox logistic regression analysis is one of the commonly used methods for selecting independent prognostic factors. Unlike traditional stepwise regression, LASSO regression can process all independent variables at the same time instead of stepwise. This improvement dramatically increases the stability of modeling. We used the LASSO logistic regression method to avoid overfitting in the case of small samples. We screened the clinical characteristics that are most relevant to the OS of childhood ALL using the R software “glmnet” package and constructed a regression model that includes the selected variables (17).

### Nomogram Construction and Validation

Based on the LASSO regression analysis, a nomogram was constructed using the potential risk factors identified in the training cohort. The concordance index (C-index) was used to estimate the predictive performance of the nomogram. Larger C-index values indicated higher predictive accuracy of the model. The calibration plot was used to compare the observation probability and prediction probability of the nomogram. The area under the receiver operating characteristic curve evaluated the predictive accuracy of the nomogram for 3-, 5-, and 10-year OS. By comparing the nomogram plot predictions with the observed Kaplan–Meier estimates, the training and validation cohorts were used to validate the nomogram, respectively. Additionally, the R software was used to analyze the Cox proportional hazards model of high-risk factors and evaluate the association between the survival time of high-risk patients and the predictors.

### Data Processing and Statistical Analysis

The categorical variables were described as counts and percentages, while continuous variables were described as the mean (or median) and range. The *t*-test and chi-squared test were used to compare continuous and categorical variables, respectively. A *P* < 0.05 denoted statistical significance. The OS was used as the primary endpoint. OS is defined as the interval from diagnosis to death or last follow-up, regardless of the cause of death. All calculations were performed using the R Environment for Statistical Computing (http://www.r-project.org/, version 3.6.1; Vienna, Austria). The R package “survival” (version 2.44-1.1), “foreign” (version 0.8-72), and “timeROC” (version 0.3) were used.

## Results

### Patient Characteristics

A total of 489 children with ALL met the inclusion criterion, and the data were randomly divided into a training group (*n* = 345) and a validation group (*n* = 144). The median OS of the training and verification cohorts was 2,678 days (range: 28–5,740 days) and 2,531 days (range: 52–5,598 days), respectively. The demographic and clinicopathological features of the training and validation cohorts are shown in Table 1. The results showed that there were no significant statistical differences between the two groups of characteristic variables (*P* > 0.05).

**Table 1:** Demographics and clinicopathologic characteristics of patients with acute lymphocytic leukemia.

**Demographic or characteristic**	**Training cohort** **(*n* = 345)**		**Validation cohort** **(*n* = 144)**		***P*- value**
	**Patients (*n*)**	**%**	**Patients (*n*)**	**%**	
Gender					
Male	199	57.7	80	55.6	0.369
Female	146	42.3	64	44.4	
Race					
Black and others	27	7.8	15	10.4	0.408
Asian	18	5.2	7	4.9	
White	300	87.0	122	84.7	
Age, days					
≤ 1986	114	33.1	48	33.3	0.562
1987–4253	77	22.3	24	16.7	
> 4253	154	44.6	72	50.0	
WBC (10^9^/L)					
≤ 41.0	171	49.6	76	52.8	0.766
41.0–200	130	37.7	48	33.3	
> 200	44	12.7	20	13.9	
CNS status					
CNS 1	267	77.4	115	79.9	0.330
CNS 2	53	15.4	23	16.0	
CNS 3	25	7.2	6	4.1	
BM relapse					
Yes	111	32.2	53	36.8	0.345
No	234	67.8	91	63.2	
CNS relapse					
Yes	40	11.6	18	12.5	0.761
No	305	88.4	126	87.5	
Testes relapse					
Yes	2	0.6	1	0.7	0.650
No	343	99.4	143	99.3	
ETV6/RUNX1 fusion status					
Yes	25	7.2	11	7.6	0.851
No	320	92.8	133	92.4	
TRISOMIES 4/10 status					
Yes	22	6.4	11	7.6	0.693
No	323	93.6	133	92.4	
MLL status					
Yes	21	6.1	9	6.2	0.545
No	324	93.9	135	93.8	
TCF3 PBX1 status					
Yes	44	12.8	17	11.8	0.881
No	301	87.2	127	88.2	
BCR ABL1 status					
Yes	9	2.6	2	1.4	0.520
No	336	97.4	142	98.6	
Vital status					
Alive	244	70.7	93	64.6	0.199
Dead	101	29.3	51	35.4	
OS (days)					
Median	2 678	2 531	0.356		
Range	28–5 740	52–5 598			

*WBC, white blood cell counts at diagnosis; CNS status, central nervous system status at diagnosis; BM relapse, bone marrow site of relapse; CNS relapse, central nervous system site of relapse; Testes relapse, testes site of relapse; MLL, mix lineage leukemia; OS (days), overall survival time in days*.

### Determination of the Optimal Cut-Point

**Figure 1:**
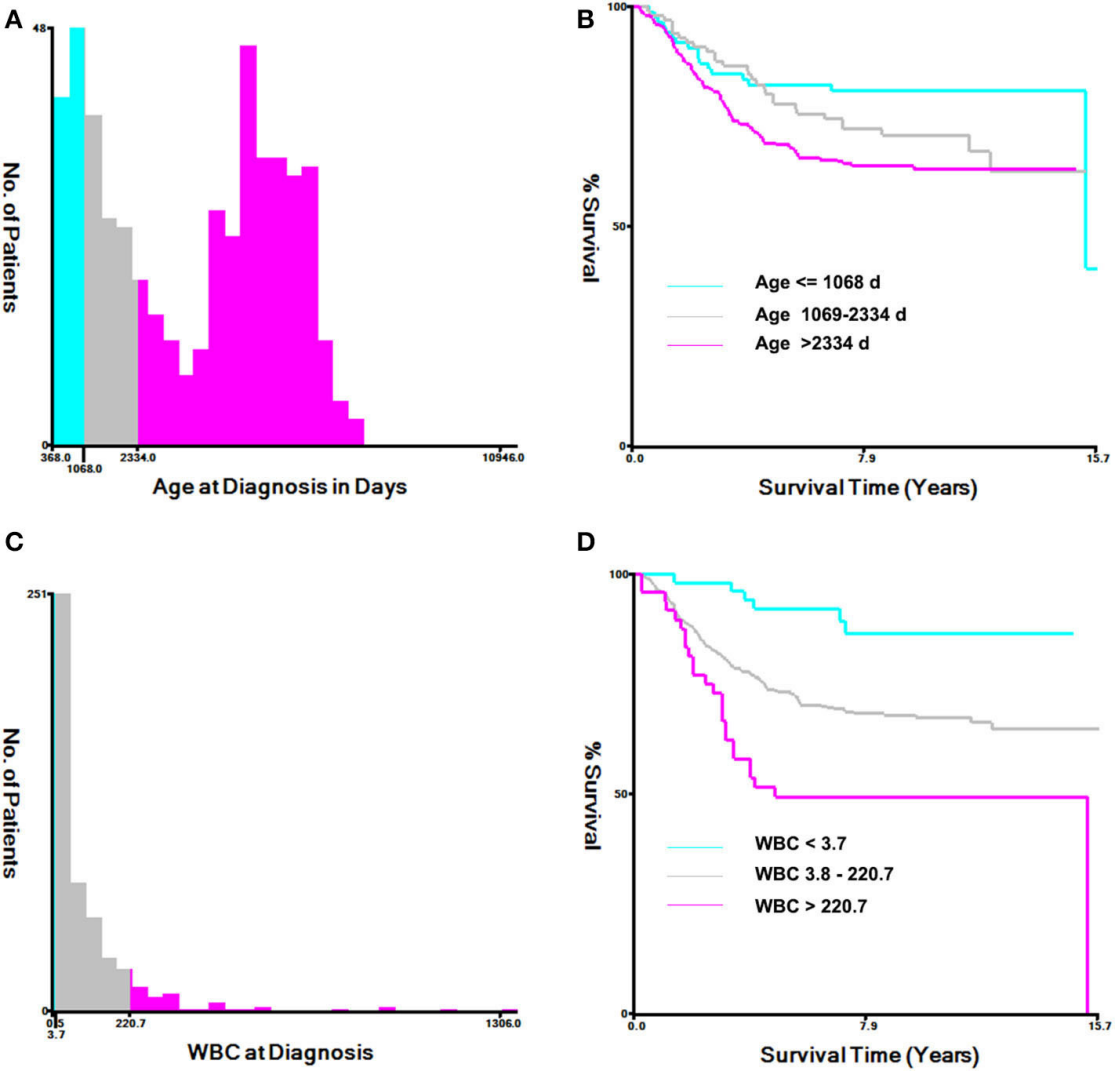
Analysis of age at diagnosis and peripheral white blood cell count (WBC) using the X-tile software. **(A)** X-tile determined the cut-point of age and divided patients into high- (>2,334 days days), medium- (>1,069–2,334 days), and low-(≤1,068 days) risk groups. **(B)** Kaplan–Meier survival curves for overall survival according to age stratification. **(C)** X-tile determined the cut-point of WBC and divided patients into high- (>220.7 × 10^9^/L), medium- (3.8–220.7 × 10^9^/L), and low- (≤3.7 × 10^9^/L) risk groups. **(D)** Kaplan–Meier survival curves for overall survival, according to WBC stratification.

The histogram and Kaplan–Meier survival curve stratified by age and WBC are shown in Figure 1. The ages were divided into three groups: low (≤1,068 days), medium (1,069–2,334 days), and high (>2,334 days) (Figure 1A). Compared with the low group, the survival times of the medium group and the high group were significantly decreased (*P* < 0.001, Figure 1B). Similarly, WBC counts were also divided into three groups: low (≦3.7 × 10^9^/L), medium [(3.8–220.7) × 10^9^/L], and high (>220.7 × 10^9^/L) (Figure 1C), As seen from Figure 1D, the survival times of the two groups with high WBC were significant differences from that of the low group (*P* < 0.0001, Figure 1D).

### LASSO Regression Analysis

**Figure 2:**
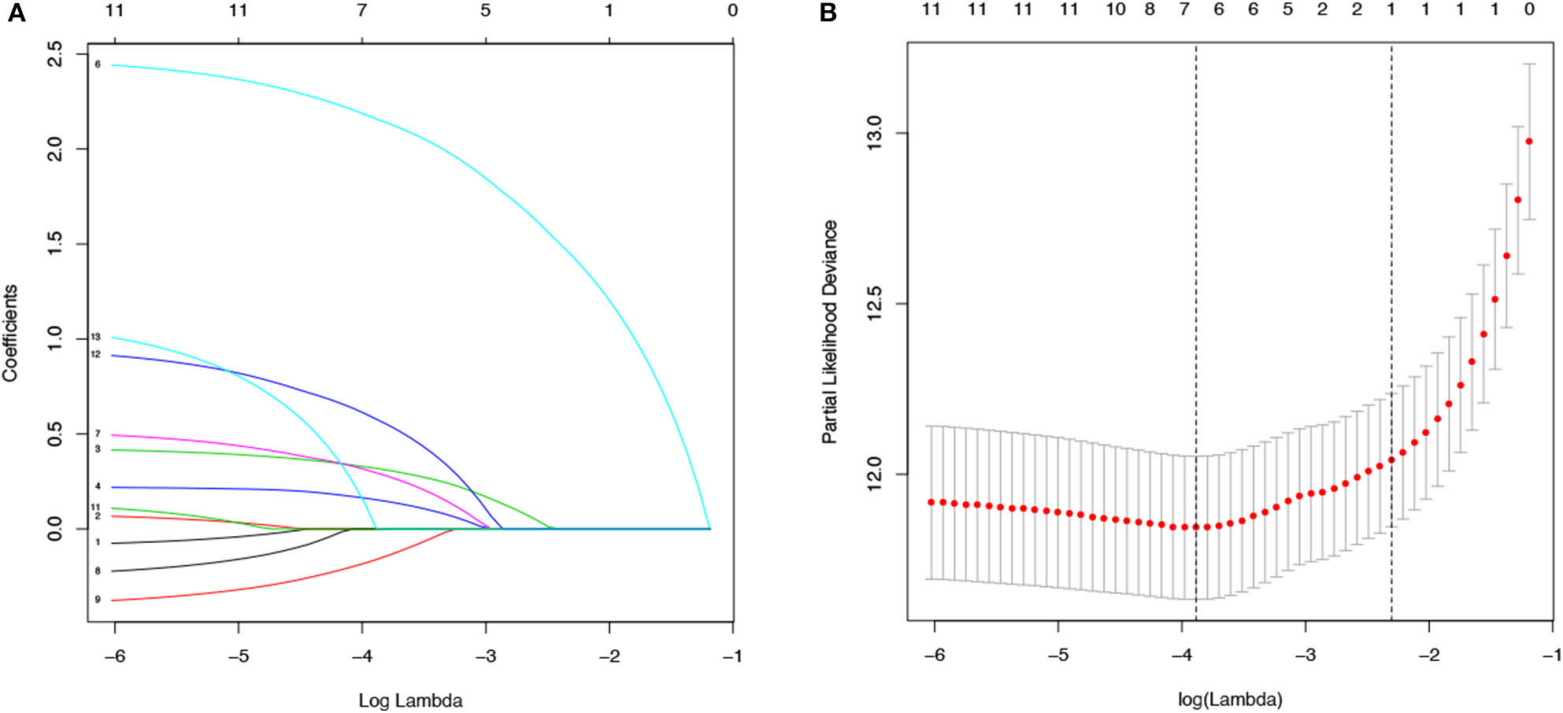
LASSO regression for the selection of characteristic parameters. **(A)** Penalty graph of thirteen characteristic variable coefficients. With the change of the penalty coefficient lambda, an increasing number of variable coefficients are compressed; finally, most of the variable coefficients are compressed to zero. **(B)** In the LASSO logistic regression model, the best penalty coefficient lambda was selected using a 10-fold cross-validation and minimization criterion. The best lambda was selected at the lowest point of the curve (lambda = 7). Seven variables with non-zero coefficients were selected at the best lambda, and the feature parameters without information are removed to realize the automatic selection of feature parameters.

**Figure 3:**
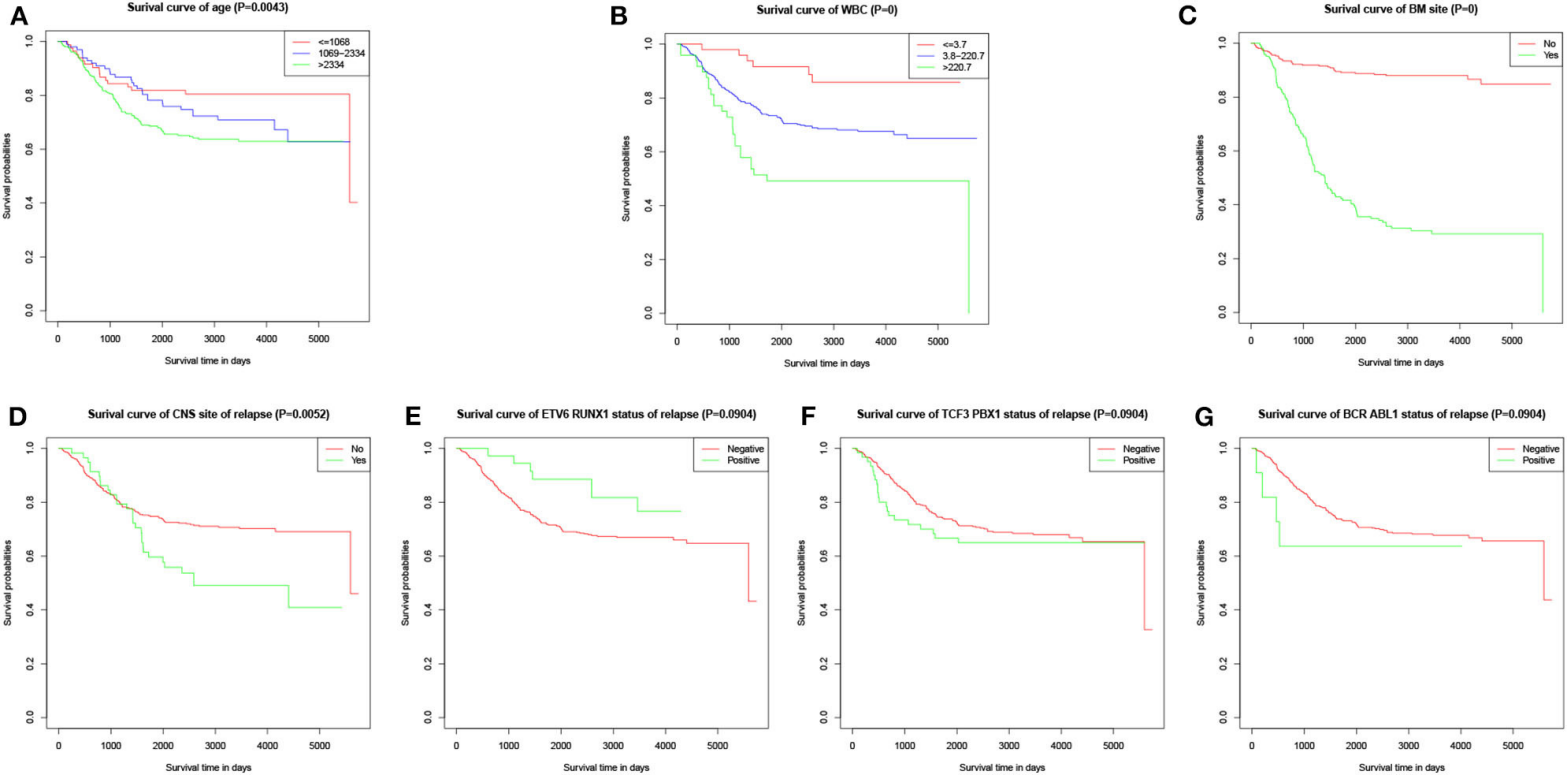
Kaplan–Meier survival curves of characteristic parameters in the training cohort. **(A)** Kaplan–Meier survival curves of age at diagnosis. **(B)** Kaplan–Meier survival curves of peripheral WBC at diagnosis. **(C)** Kaplan–Meier survival curves of bone marrow site of relapse. **(D)** Kaplan–Meier survival curves of CNS site of relapse. **(E)** Kaplan–Meier survival curves of ETV6/RUNX1 fusion status. **(F)** Kaplan–Meier survival curves of TCF3/PBX1 fusion status. **(G)** Kaplan–Meier survival curves of BCR/ABL1 fusion status.

The values with the smallest error were selected using the cross-validation method in the LASSO regression model. All collected clinical data and selected characteristics (*n* = 16) input surrogate models were refitted (Figure 2A). LASSO compresses the coefficients of most covariates to 0, leaving 11 non-zero coefficients. Variables with non-zero coefficients are screening variables (Figure 2B). According to Figure 2B, the variables reaching the minimum value of partial likelihood deviance were 7, and it could be seen from Figure 2A that the variables corresponding to the seven colored-line that intersect the red dotted line were the model building variables. Finally, seven variables, including age at diagnosis, peripheral white blood cells, bone marrow and CNS site of relapse, ETV6/RUNX1 fusion, TCF3/PBX1, and BCR/ABL1 status, were screened as independent high-risk factors. The corresponding Kaplan–Meier survival curves were shown in Figures 3A–G. Furthermore, the Cox regression analysis of selected characteristic variables was shown in Table 2.

**Table 2:** Cox regression analysis of variables associated with survival for patients with acute lymphocytic leukemia.

**Variables**	**Univariate**			**Multivariate**		
	**Hazard ratio**	**95% CI**	***P*-value**	**Hazard ratio**	**95% CI**	***P*-value**
Gender						
Female	–	Reference		–	Reference	
Male	0.999	0.724–1.378	0.994	1.170	0.835–1.639	0.363
Age, days						
≤ 1068	–	Reference		–	Reference	
1069–2334	1.470	0.807–2.679	0.208	1.809	0.960–3.409	0.067
2334	2.001	1.193–3.355	0.009^*^	2.615	1.515–4.512	<0.001^*^
Race						
Black of African American or others	–	Reference		–	Reference	
Asian	0.341	0.115–1.012	0.047^*^	0.053	0.112–1.083	0.068
White	0.681	0.411–1.130	0.212	0.137	0.437–1.359	0.368
WBC at diagnosis, × 10^9^/L						
≤ 3.7	–	Reference		–	Reference	
3.8-220.7	2.928	1.290–6.646	0.010^*^	2.230	1.001–5.264	0.048^*^
> 220.7	5.611	2.299–13.70	<0.001^*^	2.971	1.159–7.616	0.023
CNS status at diagnosis						
CNS 1	–	Reference		–	Reference	
CNS 2	1.434	0.957–2.158	0.074	1.409	0.906–2.190	0.128
CNS 3	0.584	0.257–1.327	0.199	0.793	0.340–1.848	0.591
Bone marrow site of relapse						
No	–	Reference		–	Reference	
Yes	8.241	5.705–11.91	< 2e−16^*^	10.02	6.687–15.02	< 2e−16^*^
CNS site of relapse						
No	–	Reference		–	Reference	
Yes	1.768	1.179–2.652	0.006^*^	1.252	0.819–1.914	0.299
Testes site of relapse						
No	–	Reference		–	Reference	
Yes	1.951	0.483–7.879	0.348	0.691	0.166–2.880	0.612
ETV6 RUNX1 status						
No	–	Reference		–	Reference	
Yes	0.525	0.246–1.122	0.096	0.598	0.264–1.353	0.217
TRISOMIES status						
No	–	Reference		–	Reference	
Yes	0.636	0.298–1.359	0.243	0.634	0.285–1.412	0.265
MLL status						
No	–	Reference		–	Reference	
Yes	0.967	0.491–1.905	0.924	1.227	0.589–2.556	0.586
TCF3 PBX1 status						
No	–	Reference		–	Reference	
Yes	1.186	0.747–1.881	0.469	1.843	1.101–3.086	0.020^*^
BCR ABL1 status						
No	–	Reference		–	Reference	
Yes	1.451	0.537–3.917	0.463	6.684	1.144–3.208	0.001^*^

* *P-value <0.05. CI, confidence interval; WBC, white blood count; CNS, central nervous system*.

### Prognostic Nomogram for OS

**Figure 4:**
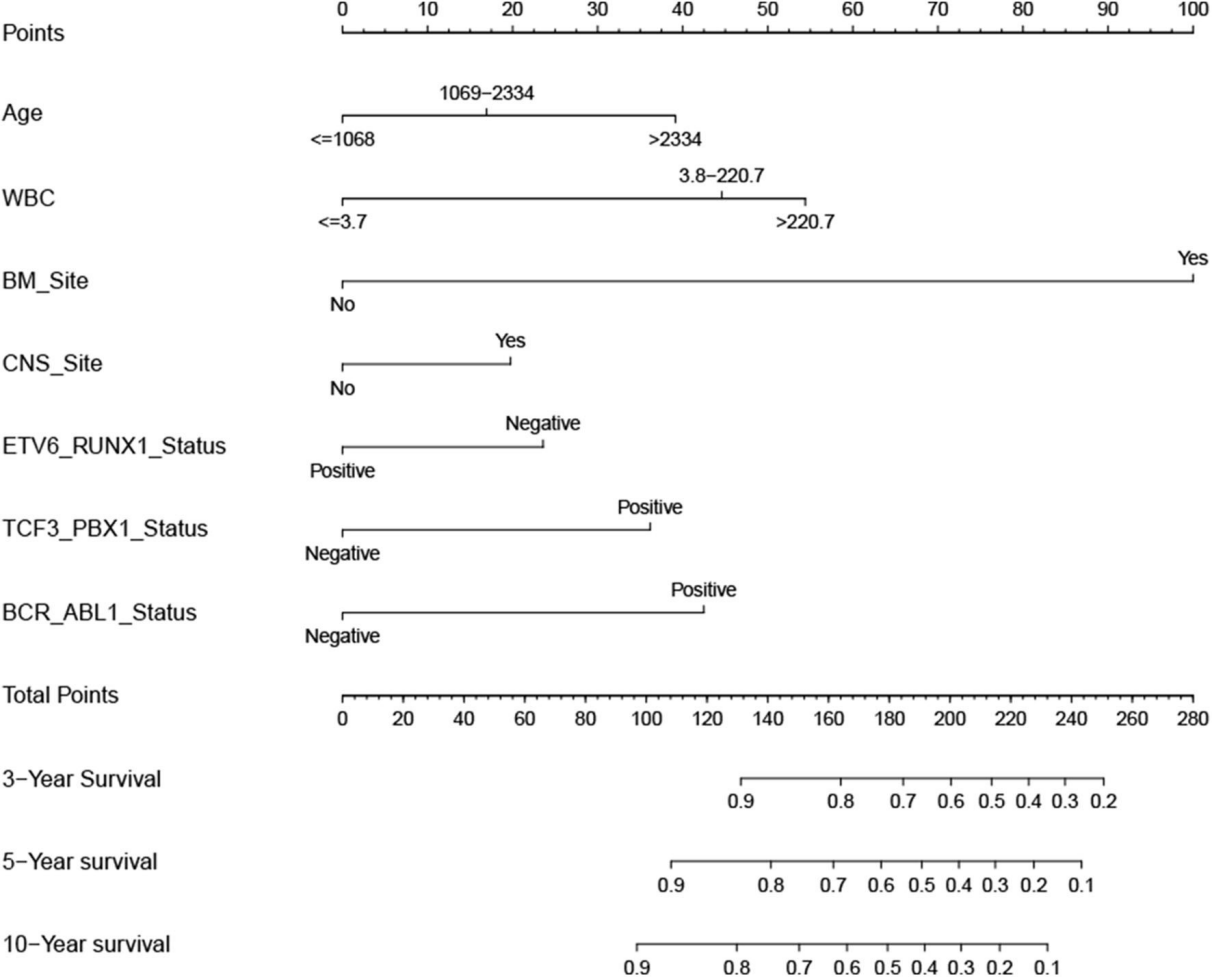
Overall survival predictive nomogram for children with acute lymphoblastic leukemia. To use the nomogram, the value of an individual patient is located on each variable axis, and a line is drawn upward to determine the number of points received for each variable value. The sum of these numbers is located on the Total Points axis, and a line is drawn downward to the survival axes to determine the likelihood of 3-, 5-, or 10-year survival.

**Figure 5:**
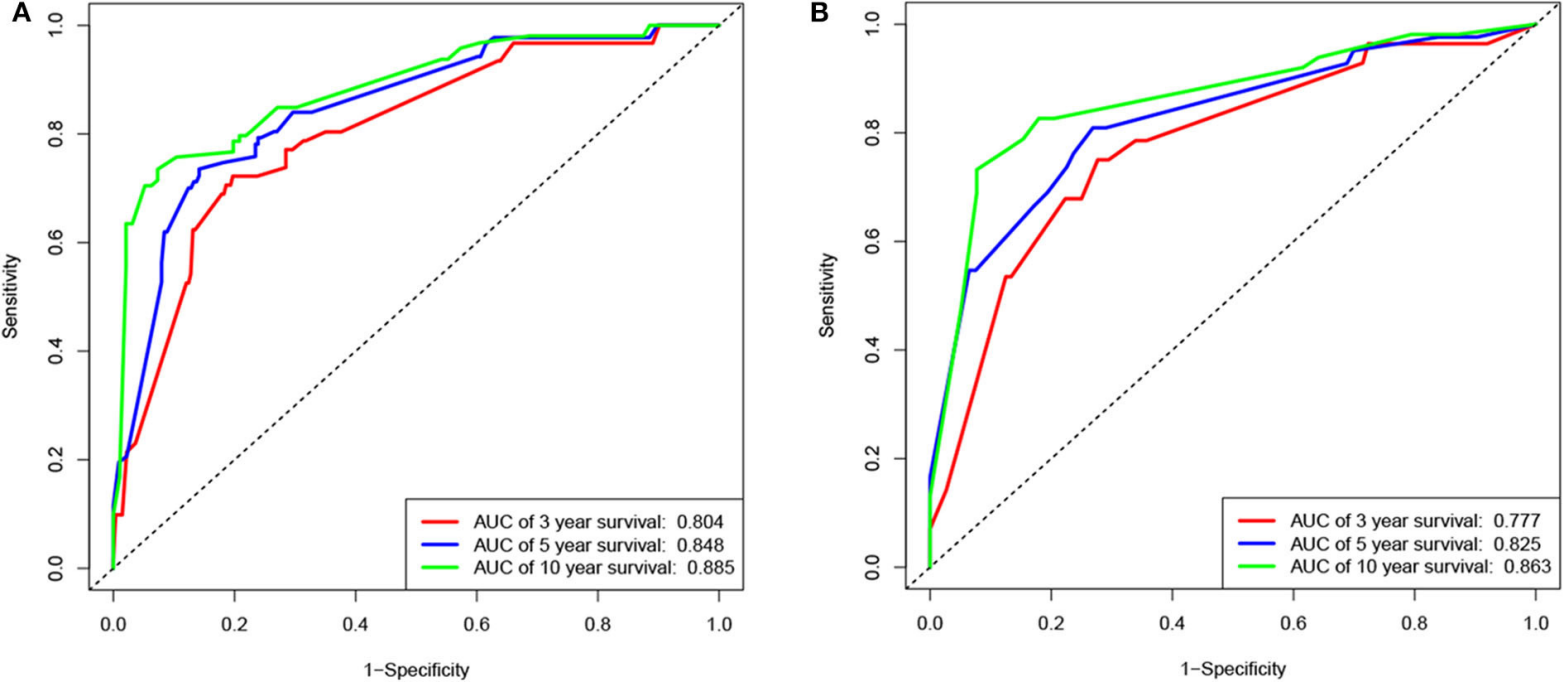
Receiver operating characteristic (ROC) curves of the nomogram for the training and validation cohorts. ROC curves for predicting 3-, 5-, or 10-year survival in the training **(A)** and validation **(B)** cohorts.

**Figure 6:**
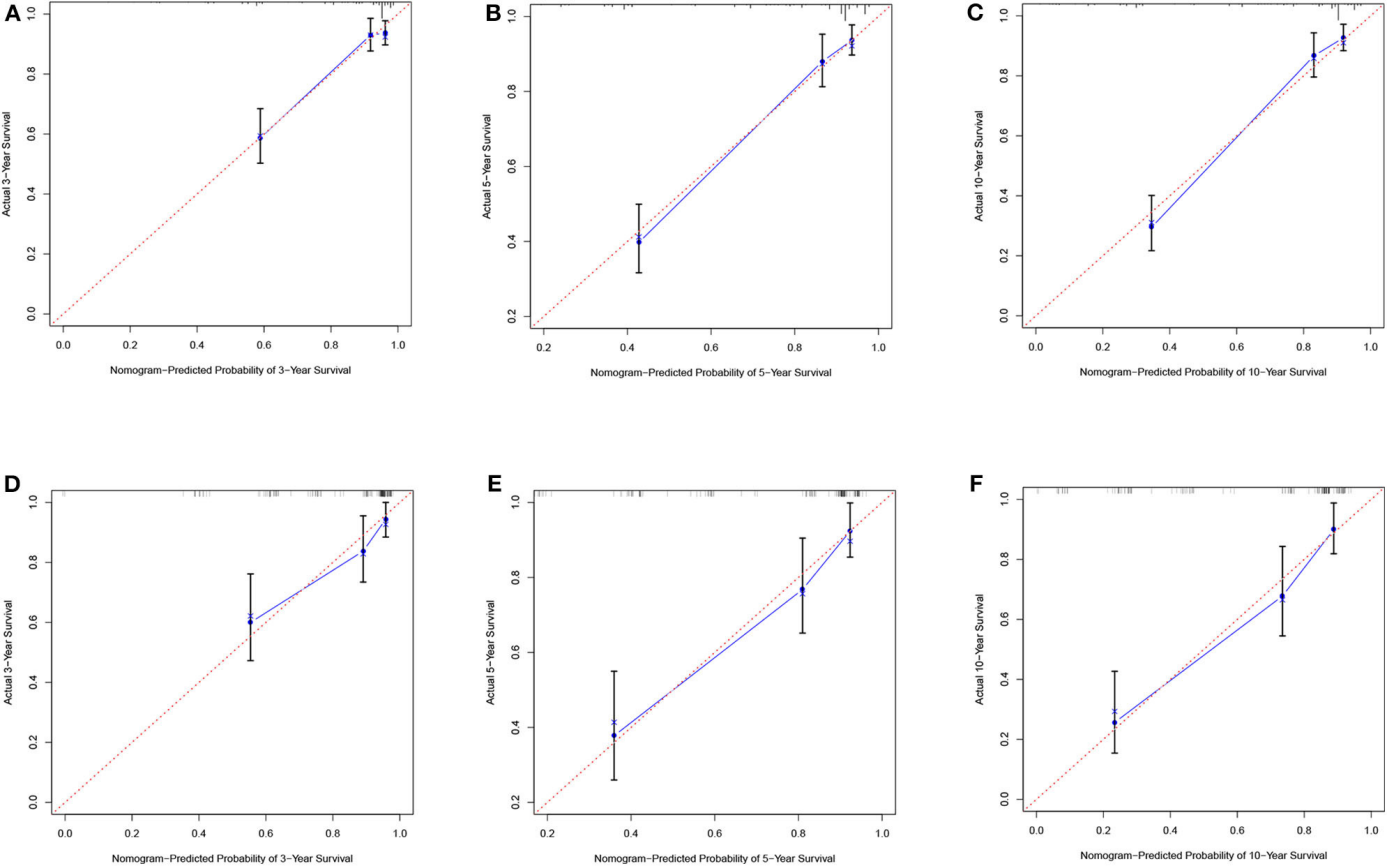
Calibration plots of the nomogram for the training and validation cohorts. Calibration plot of observed and predicted probabilities of the nomogram for the training **(A–C)** and validation **(D–F)** cohorts at 3, 5, or 10 years, respectively.

According to the results of the LASSO regression analysis, we integrated the factors, such as age at diagnosis, WBC, bone marrow site of relapse, CNS site of relapse, ETV6/RUNX1 fusion, TCF3/PBX1, and BCR/ABL1 status. We subsequently used the training cohort to establish a prognostic nomogram graph for children with ALL (Figure 4). The C-index of the prognostic nomogram was 0.809 (95% confidence interval: 0.766–0.852), and the receiver operating characteristic curves of the nomogram exhibited good discrimination for predicting the prognosis of ALL in children with ALL. The area under the receiver operating characteristic curve values of the nomogram for the 3-, 5-, and 10-year OS were 0.804, 0.848, and 0.885, respectively (Figure 5A). Furthermore, the calibration curve showed good agreement between the predicted and observed values in the 3-, 5-, and 10-year OS (Figures 6A–C). Using this nomogram, we can quantitatively predict the OS of patients at 3, 5, and 10 years based on their initial diagnosis and response after treatment.

### Validation of the Prognostic Nomogram

In the validation cohort, the C-index was 0.826 (95% confidence interval: 0.767–0.885). Moreover, there was no statistically significant difference in the prognostic nomogram C-index between the training and validation cohorts (*P* > 0.05). As shown in Figure 5B, the area under the receiver operating characteristic curve values for the 3-, 5-, and 10-year OS in the validation cohort were 0.777, 0.825, and 0.863, respectively. Similarly, the calibration curve also showed good agreement between the predicted and observed values for the 3-, 5-, and 10-year OS (Figures 6D–F).

### Analysis of Risk Scores Based on High-Risk Factors

**Figure 7:**
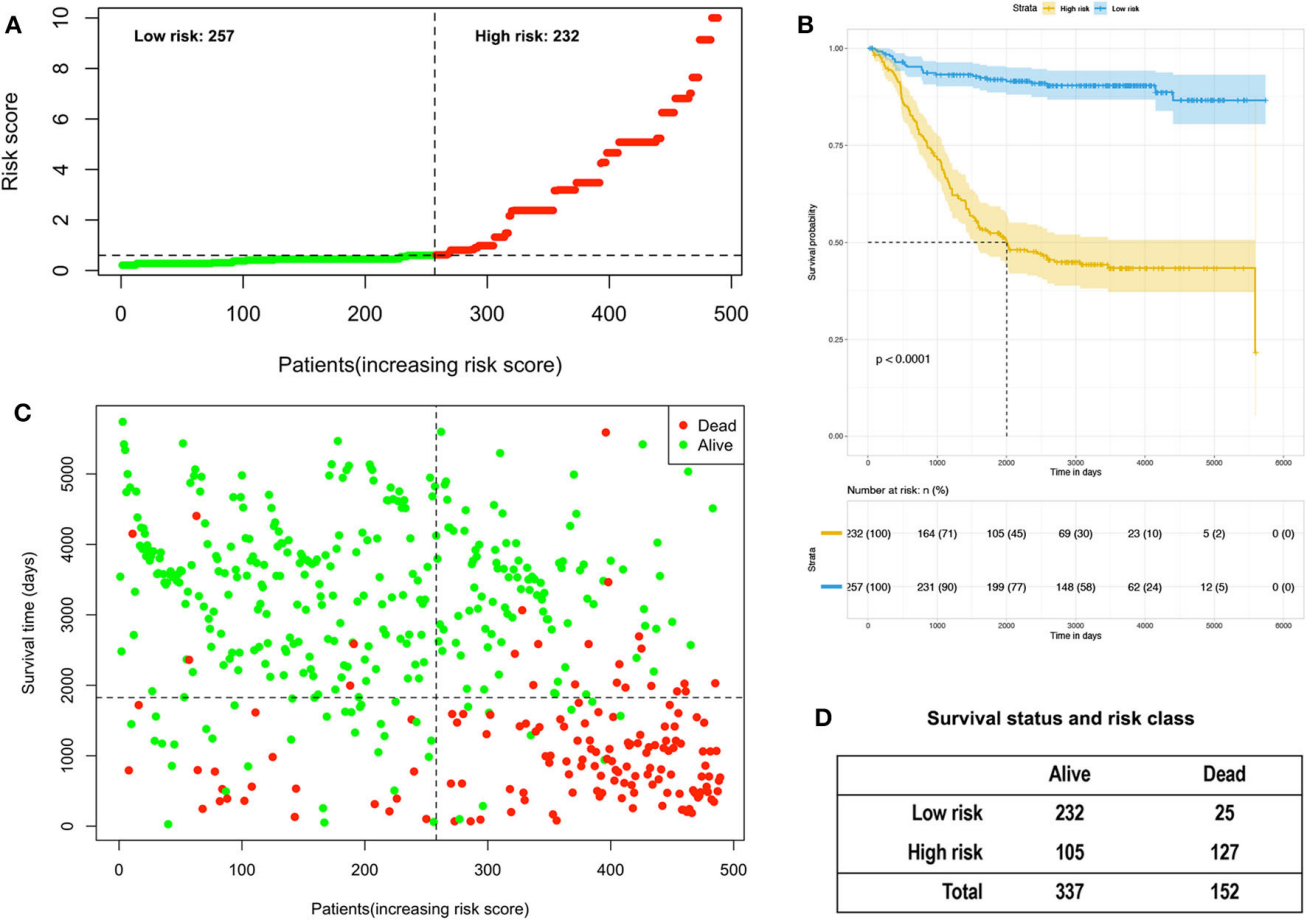
Analysis of the risk score of children with acute lymphoblastic leukemia in the training and validation group. **(A)** Distribution of risk scores based on seven clinical characteristics. **(B)** Survival status and duration of disease in children with acute lymphoblastic leukemia; **(C)** Kaplan–Meier survival curves based on high- and low-risk scores. **(D)** Survival status and risk level of all patients.

A Cox proportional hazards model was constructed based on the high-risk factors in the nomogram to analyze the association between patient survival time and predictive indicators according to the risk score. Based on the risk score curve (Figure 7A), the children were divided into low-risk (*n* = 257) and high-risk groups (*n* = 232). According to the survival status curve, most of the risk scores were short-lived High (Figure 7B). Among the dead children, there were 127 high-risk cases, accounting for 83.6% (127/152); of the surviving children, there were 232 low-risk cases, accounting for 68.8% (232/337) of the total (Figure 7D). Notably, the calculated risk score correlated with OS (*P* < 0.01) (Figure 7C).

## Discussion

In this study, we performed LASSO regression analysis using public data from 489 cases of childhood ALL. Different from traditional linear regression, LASSO regression is to perform variable selection and regularization while fitting a generalized linear model. While ensuring the best fitting error, it makes the parameters as simple as possible, making the model's generalization ability Stronger. Since there are many variables affecting children's ALL prognosis, the number of non-zero coefficients in the model is determined by LASSO regression analysis (Figure 2A), combined with the λ value corresponding to the mean of the minimum target parameter to filter the variables (Figure 2B), and selectively put the seven variables into the model to obtain better model performance parameters. Therefore, this good characteristic of LASSO regression has been widely used in data mining research by many researchers (18, 19). The article identified seven risk factors, namely age at diagnosis, WBC, bone marrow site of relapse, CNS site of relapse, ETV6/RUNX1,TCF3/PBX1, and BCR/ABL1 status. Subsequently, a nomogram of childhood ALL, including these factors, was established to accurately predict the OS of children with ALL. This approach provided a convenient evaluation tool and personalized treatment for childhood ALL based on different high-risk factors.

The bone marrow site of relapse was the most common recurrence factor for childhood ALL. In the analysis, the recurrence rate of bone marrow reached 33.5% (164/489 cases), which was consistent with the data obtained from the American Children's Cancer Cooperative Group study (20). Of note, the bone marrow site of relapse exhibited the most significant score in the nomogram (score = 100), indicating that it was the most significant factor for poor prognosis. This conclusion is consistent with those reported in numerous studies (20–24). The mesenchymal cells in the bone marrow microenvironment secrete a large number of bioactive substances to promote angiogenesis and provide sufficient nutrients for leukemic cells (25, 26). Moreover, the extracellular matrix, mesenchymal cells, and leukemic cells could interact with each other to generate anti-apoptotic signals, ensure the continued existence of drug-resistant leukemic cells, and provide conditions for the promotion of leukemia (25, 26). This may explain the higher proportion of bone marrow recurrence vs. other sites and the poor prognosis of children with ALL. Therefore, medical management should also focus on the prevention of bone marrow recurrence in children with ALL. Hematopoietic stem cell transplantation should be performed as appropriate to improve the OS of children with relapses. Moreover, it is important to identify unknown genes related to relapses, with the aim to further reduce the recurrence rate and increase OS (27).

Improvement in the permeability of first-line chemotherapy drugs resulted in fewer cases of extramedullary recurrence (28). In this study, the recurrence rates of the CNS and testes sites were 11.9% (58/489 cases) and 0.6% (3/489 cases), respectively. This may be attributed to the fewer reported cases of simple extramedullary recurrence. Although recurrence in the CNS site was one of the seven prognostic factors in the nomogram, the score was the lowest among the factors (score = 18). Furthermore, testicular recurrence was not an independent prognostic factor in childhood ALL.

Fusion genes were formed by translocation and rearrangement of chromosomes and were the result of a combination of genetic and environmental factors. Many ALL-related fusion genes had been detected, which played a vital role in the pathogenesis, diagnosis, efficacy evaluation, prognosis prediction, and treatment guidance of leukemia (29). They were also an essential basis for the monitoring of minimal residual disease (MRD). In this article, ETV6/RUNX1, TCF3/PBX1, and BCR/ABL1 fusion genes were considered to be important for the prognosis of childhood ALL. The ETV6/RUNX1 fusion gene was the most common chromosomal translocation abnormality in childhood ALL. The positive rate reported in the literature was about 25% of children B-line ALL. Simultaneously, some studies had shown that the ETV6/RUNX1 fusion gene was a useful biomarker of prognosis in children B-ALL (30, 31), which was confirmed again in this article. The PBX1 gene was the most common translocation partner of TCF3, resulting in the fusion of the TCF3/PBX1 gene. It was currently classified as a recurrent genetic abnormality of B-ALL by WHO and a risk factor for the medium-risk group by the Chinese children's ALL diagnosis and treatment specification (32, 33). The BCR-ABL1 fusion gene was formed by the translocation of t_(9, 22)(q 34; q 11)_, also known as the Philadelphia chromosome (Ph chromosome). The National Comprehensive Cancer Network (NCCN) classified BCR-ABL1 fusion gene-positive patients as a low-risk group. In 2018, the Chinese children's ALL diagnosis and treatment norms used the BCR-ABL1 fusion gene as a risk factor for poor prognosis. Previous studies and the results in this article confirmed that fusion genes are important molecular biological markers for ALL prognosis. Clinically, appropriate detection techniques should be selected to improve the detection rate of fusion genes, significantly improve the prognosis and the quality of life children.

Consistent with previous studies, age was also an important prognostic factor for childhood ALL in this research. However, most of the previous literature divided the age into groups of <1 year (365 days), 1–10 years (365–3,650 days), and ≥ten years (≥3,650 days), according to the Children's Oncology Group (27–29). In this study, the age was divided into three groups: ≤1,068 days (≤3.0 years), 1,069–2,334 days (3.0–6.4 years), and ≥2,334 days (≥6.4 years) based on the cut-point optimization of the OS. In previous studies, the association between age grouping and survival outcomes was not evaluated, which may be responsible for the different conclusions reported between studies. This study found that children aged ≤1,068 days (≤3.0 years) had a better prognosis, While the age increases, the prognosis gradually deteriorates. This result was similar to the findings reported by Tai et al. (12). However, the German BFM Collaboration Group and the Chinese CCLG-ALL-2008 program (34, 35), stated that children aged <1 and ≥10 years had a poor prognosis. Differences in survival between age groups may be due to the DNA index and specific chromosomal rearrangements, or variations in the amount of data included and the immunophenotype among studies (36, 37). Similarly, the Children's Oncology Group classified the WBC at diagnosis into two groups (≤50 × 10^9^/L and >50 × 10^9^/L) (27–29). According to the cut-point optimization, the WBC counts of patients in this study were divided into ≤3.7 × 10^9^/L, (3.8–220.7) × 10^9^/L, and >220.7 × 10^9^/L. However, these studies concluded that the high WBC at diagnosis was a risk factor for children with ALL, only their cut-points differed, which may be due to differences in race, genetic background, and other factors (38). According to the literature (16), the cut-point should be optimized for continuous variables, such as age and WBC. Therefore, for the two important risk factors of age and WBC, their optimal cut-off point still requires a multi-center, large-sample cohort study.

For clinical applications, it was essential to identify risk factors as efficiently as possible. We constructed a nomogram using the TARGET dataset to predict the overall survival of children with ALL. The variables required for the nomogram constructed in this study were standard in clinical practice and were easily available. The risk scores based on these seven variables could effectively distinguish between high-risk and low-risk children and could quickly assist clinical decision-making, which had significant clinical value for improving children's prognosis. However, this study had some limitations. Firstly, it was the lack of verification of external data. The training and validation cohorts in this study were derived from the same TARGET dataset, which could overfit the model. Therefore, it should be ensured that multi-center verification with a large sample size could provide advanced evidence for clinical application. Secondly, the sample size in the article was relatively small. The samples finally included in the analysis were only half of the total samples because of the most samples in TARGET without genetic markers. Although the seven variables selected in this article were approved by numerous literature, the nomogram composed of them still needs more data to verify and improve in the future continuously.

## Data Availability Statement

The datasets generated for this study are available on request to the corresponding author.

## Author Contributions

DZ, YC, and YG designed this research and analyzed and interpreted the data. YG, ZjZ, and JF mainly developed methodology. DZ, YC, JY, ZhZ, and YT collected data and performed preprocessing. DZ, YC, and YG were significant contributions in writing the manuscript. All authors read and approved the final manuscript.

## Conflict of Interest

The authors declare that the research was conducted in the absence of any commercial or financial relationships that could be construed as a potential conflict of interest.
